# Alternative treatment for recurrent keloids: initial clinical experience with Rhenium-188 using a specialized device

**DOI:** 10.1007/s00259-025-07184-4

**Published:** 2025-03-12

**Authors:** Kgomotso M. G. Mokoala, Linda Nonjola, Thabiso Moeng, Cecilia Corbett, Martin Magwaza, Gerhard Dahlhoff, Shannon Brown, Nicholas Vetter, Mariza Vorster, Mike Machaba Sathekge

**Affiliations:** 1 Department of Nuclear Medicine, University of Pretoria & Steve Biko Academic Hospital, Pretoria, South Africa; 2 Nuclear Medicine Research Infrastructure (NuMeRI), Pretoria, South Africa; 3 Oncobeta Africa and Tautomer Biosciences, Pretoria, South Africa; 4 Oncobeta GmbH, Munchen, Munich, Germany; 5 Inkosi Albert Luthuli Central Hospital, University of Kwazulu Natal, Durban, South Africa

**Keywords:** ^188^Re, Keloids, Rhenium SCT, Single session therapy

## Abstract

**Introduction:**

Keloids have proved challenging to manage with various therapies providing variable success rates and recurrences. Alternative therapies or a multimodal approach is often necessary to ensure complete eradication and prevent recurrence. The use of radioactive creams or patches embedded with Holmium-166, Phosphorus-32 for superficial skin lesions has been documented to be safe and effective. The use of Rhenium-188 has proved effective in non-melanoma skin cancers. We report on the initial experience with Rhenium-188 SCT in the treatment of recurrent keloid lesions.

**Methods:**

Patients with recurrent keloid lesions were recruited for therapy with Rhenium-188. These patients had failed most forms of therapy including surgery, intralesional steroids and radiation therapy. Treatment with ^188^Re via a specialized unit (Rhenium SCT -Oncobeta) was applied onto the keloid lesion. A personalized treatment time was calculated for every patient. Topical ^188^Rhenium delivered as a jelly like matrix containing an insoluble dirhenium-heptasulfide was applied to every target lesion in a single session. The goal is to deliver 30 Gy to the deepest part of the lesion per session (3 mm). Patients were followed up at 2 weeks, 1, 3, 6 and 12 months for side effects as well as clinical and cosmetic outcomes.

**Results:**

A total of 58 lesions were treated. Majority of the lesions were in the head and neck region. The smallest area for treatment was 0.25cm^2^ and the largest area treated was 46.25cm^2^. With the exception of four patients (2 sessions to the same lesion), all the other patients received a single session of therapy. The mean activity administered was 256,7MBq (range: 35MBq– 663,50MBq). The treatment time averaged 350.89 min (range: 85–1304 min). There was complete response in 72% of the lesions. Hypopigmentation was the commonest expected long term side effect. After a median follow-up period of 37 months (range: 7–53), there was a 7% recurrence rate.

**Conclusion:**

Treatment with ^188^Re is a great alternative in patients with keloids that have had minimal success with other therapies. The use of the specialized applicator system provides great flexibility, reduced morbidity and great results that are comparable to other therapies.

**Clinical trial number:**

Not applicable.

## Introduction

In 1817, Alibert first introduced the term “keloid” or “cheloide” which is derived from the Greek word “chele” that means crab claw [1]. Although keloids occur in all races, Africans are more susceptible and stories of keloids are rooted in African folklore and their behaviour is well described in African proverbs and folk medicine [2]. They have a strong predilection for people of colour (hyperpigmented) and especially Africans and Asians. The prevalence in Africans has been reported to be as high as 16% although this figure may be underestimated [3].

The most frequent sites for keloids are areas of high skin tension such as neck, chest, back, ear, shoulders and beard areas [2]. Keloids range in size from small papules (few millimetres in diameter) to football size or larger [4]. The main concern with keloids is the cosmetic appeal as they can cause significant disfigurement. The other complications of these abnormal scars are pruritus, tenderness, burning, secondary infection, ulceration and restricted movement (if the keloid is in the vicinity of a joint) [5].

Management of keloids is challenging and several therapeutic strategies have been tested with variable rates of success. The best treatment strategy is prevention of the keloid. Once the scar is present, there are many treatment options to choose from. The aim of treatment should be to remove the existing growth and to prevent recurrence by inhibiting the proliferation of fibroblasts and as a result prevent the synthesis of collagen [2]. There is no universally accepted treatment modality that results in complete or permanent keloid improvement.

The use of radioactive material in the treatment of superficial skin lesions has been investigated. Pure Beta (β) emitters are preferred, as they possess ideal therapeutic characteristics of high linear energy transfer with rapid fall-off and low tissue penetration. Rhenium-188 (^188Re^) has emerged as an ideal agent in the therapy of superficial skin lesions. It has had an amazing impact in oncology with the treatment of squamous cell carcinoma and basal cell carcinoma. It provides great cosmetic results, flexibility in delivering the desired radiation dose and reduced rates of recurrence.

The use of this technology in keloids has not been thoroughly investigated. We report on the results of our initial experience with ^188Re^ SCT in the treatment of keloids.

## Methods

**Fig. 1:**
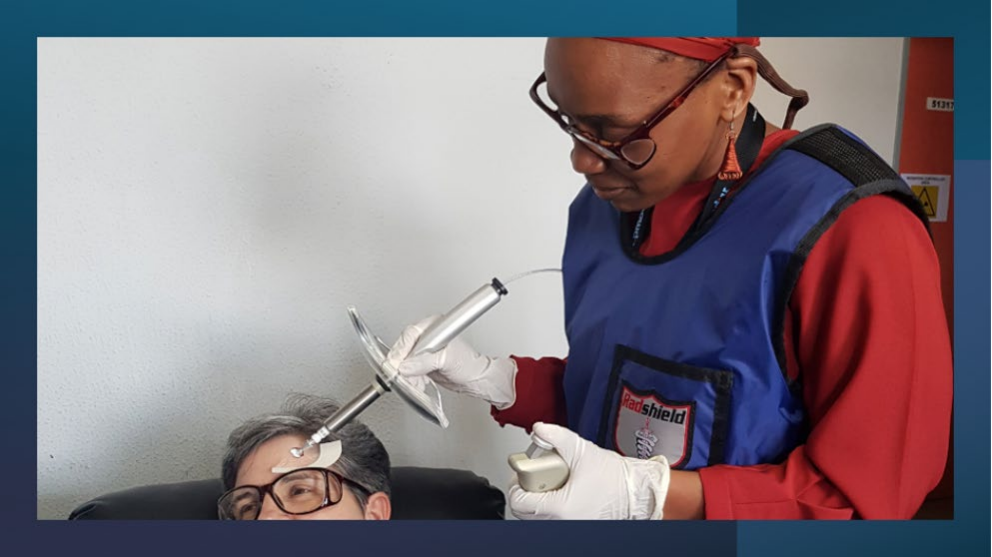
Illustration of how we use the specialized device to apply the cream onto the lesion. As described above, the area to be treated is marked with a dermatological pen, followed by application of a clear film. The Rhenium-188 cream is then applied

**Fig. 2:**
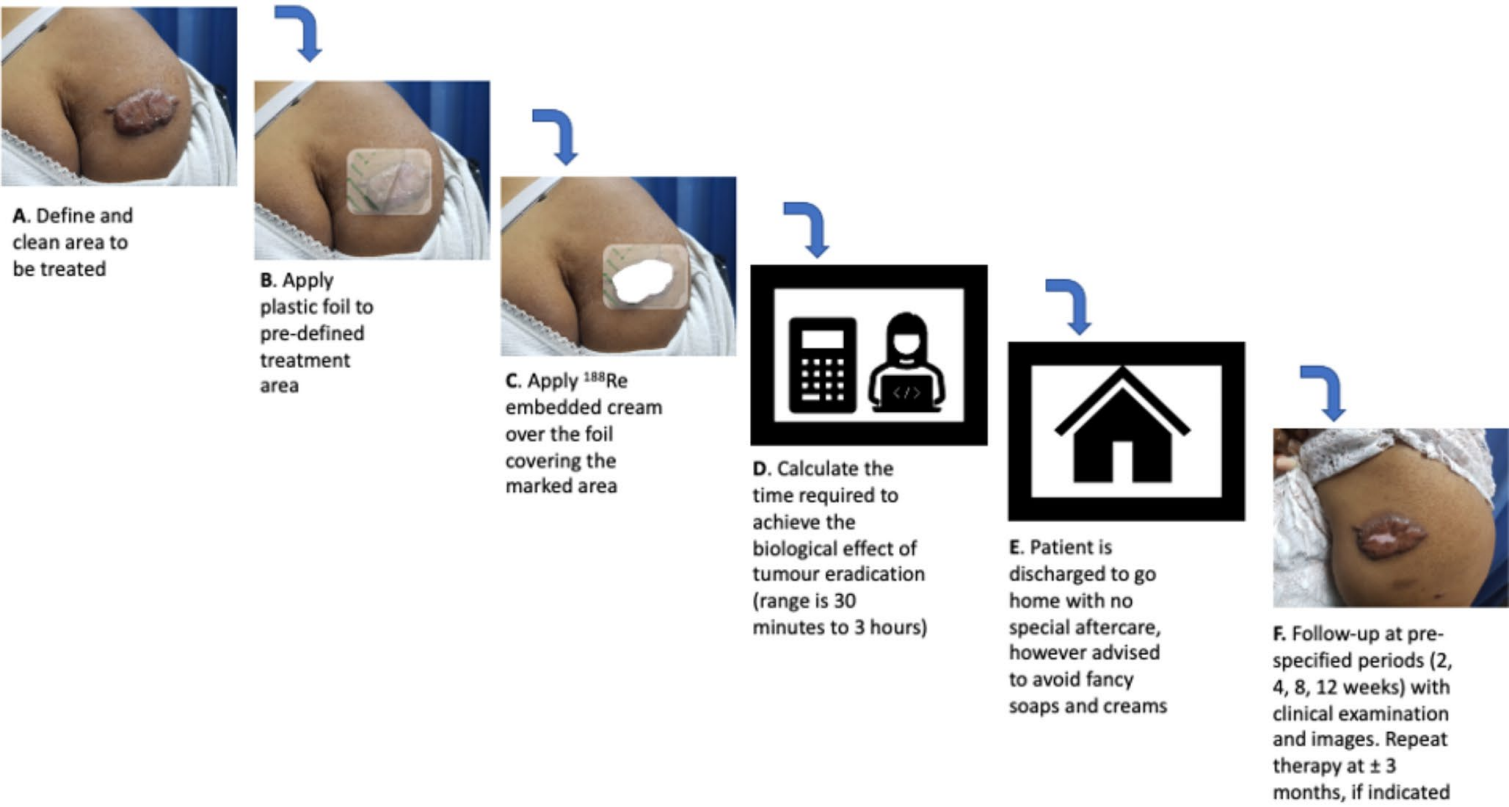
The steps of treatment on the day of therapy and the follow-up

Patients with intractable keloids that had not responded to other forms of therapy were referred to the Nuclear Medicine department from the Dermatology and Plastic surgery departments between November 2019 to October 2020. The exclusion criteria for this therapy was age less than 18 years and pregnancy in women of child bearing age. A written consent was obtained from the patients prior to therapy. Digital pictures of the lesions to be treated were taken. The lesion/s or area to be treated was marked out with a dermatological pen. Areas of scabbing or crusting were softened with saline and removed. Any areas of bleeding were stopped by applying firm pressure. The area to be treated was traced onto a graph paper in order to calculate the treatment area. A transparent protective foil was then applied onto the lesion (Fig. 1). An initial measurement of the radioactivity compound in the carpoule was made. Rhenium-188 in a gel matrix was applied onto the keloid lesion using the specially designed applicator system (Oncobeta). After application of the compound, a second measurement of the remaining activity in the carpoule was made. This was to calculate the amount of activity applied onto the keloid lesion. The VARSKIN 5 software system was used to calculate the treatment time. This time is dependent on the amount of radioactivity applied, the area and depth of the lesion to be treated. This time was also calculated with a desire to deliver 30 Gy to the deepest part of the keloid which was based on the thickness of the lesion. For post-surgical lesions / flat lesions a depth of between 1 and 2 mm was employed and for thicker lesions a depth of up to 3 mm was used. After a specified / calculated treatment time, the foil with the radioactive compound was removed, and the patients released to go home (Fig. 2).

Patients were followed-up at 2, 4, 8, 24 weeks and 12 months post-therapy. Clinical pictures were obtained before therapy as well as during follow-up. During the follow-up an assessment of the treated area was made both clinically and with measurements (rulers). The lesions were categorized as follows:

No response: < 20% reduction in height and / or diameter.

Partial response: A reduction of between 20% and 70% of the height and / or diameter.

Complete response: > 70% reduction in height and / or diameter.

Progressive disease: An increase in height or diameter of the lesion.

**Fig. 3:**
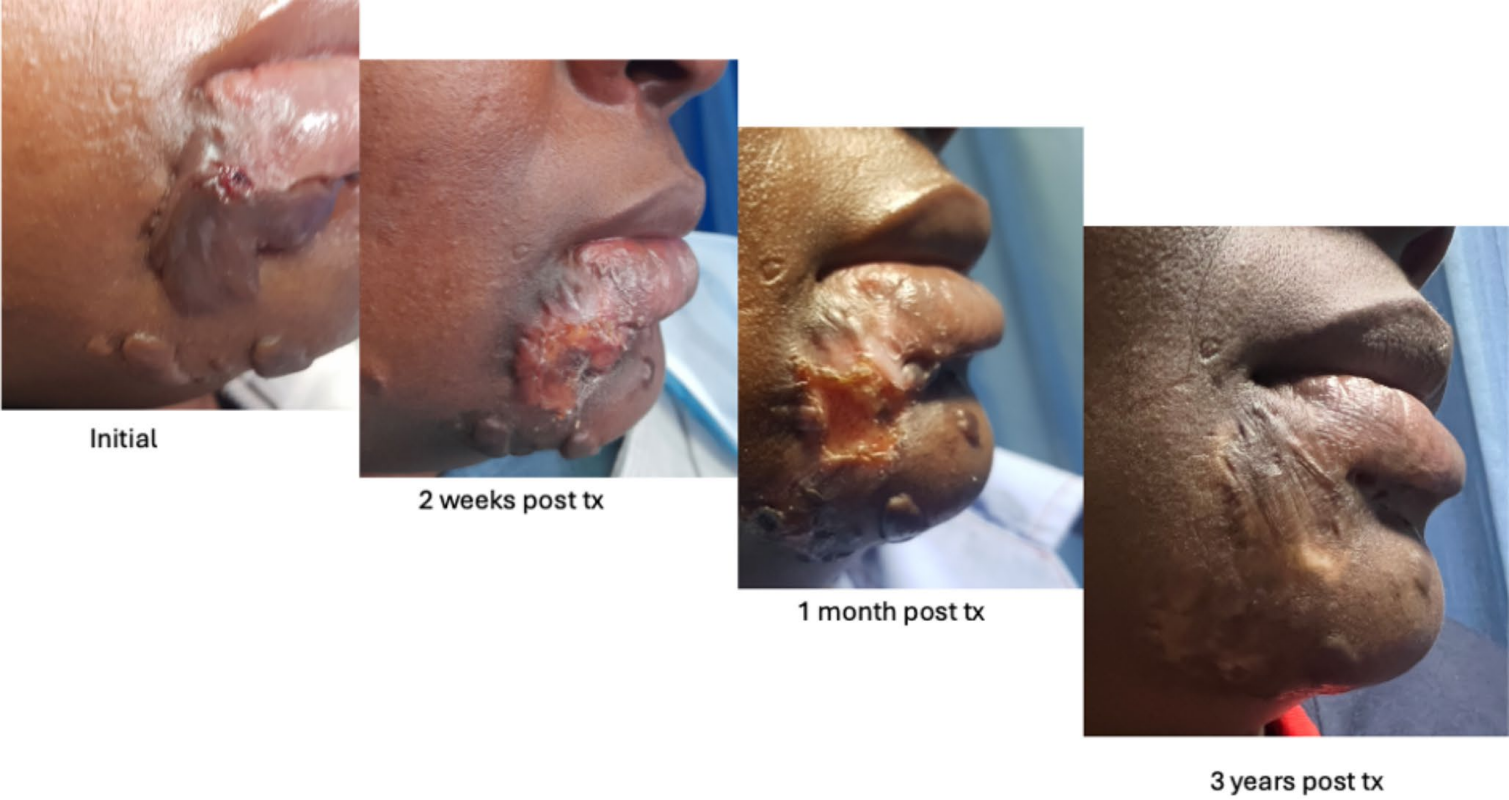
A 35 year old female with a history of spontaneous keloids peri-orally. She complained of itching and occasional sepsis, however, the major complaint was the cosmetic burden. She received a single session of 188Re with a total dose of 30 Gy and an administered activity of 217.90MBq. After a follow-up period of 36 months she had complete response

The subjective experience of patients, including their perceived relief of symptoms, were taken into account t when evaluating the treatment. All procedures performed in studies involving human participants were in accordance with the ethical standards of the institutional and/or national research committee and with the 1964 Helsinki Declaration and its later amendments or comparable ethical standards. The Research Ethics Committee of the Faculty of Health Sciences, University of Pretoria, approved the study (Reference number: 644/2023). Informed consent was obtained from all individual participants included in the study. The authors affirm that human research participants provided informed consent for publication of the images in Figs. 2 and 3.

## Results

We treated a total of 59 lesions in 29 patients (13 males and 15 females). The mean age was 40.33 years (range: 25–50 years). The race distribution was skewed towards the blacks with only one white female patient. Majority (seven) of the lesions were in the head and neck region, while the others were located on the trunk (chest and abdomen) and limbs (Table 1). The mean area for treatment was 4.65cm^2^ (range: 0.25–46.25cm^2^). The maximum permissible depth was used i.e. 3 mm for dose calculations. With the exception of four patients (2 sessions to the same lesion), all the other patients received a single session of therapy. The average activity administered was 256,7MBq (range: 35MBq– 663,50MBq). The treatment time averaged 350.89 min (range: 85–1304 min).

**Table 1:** Patient demographics and treatment details

Variable	Frequency	Percentage
**Age (years)**		
Mean ± SD	40.33 ± 12.19	
Range	25–50	
**Gender**		
Males	14	48.27
Females	15	51.73
**Race**		
White	1	3.45
Black	26	89.65
Indian	1	3.45
Mixed race	1	3.45
**Location of keloids**		
Head and neck	36	61.02
Trunk	14	23.73
Limbs	9	15.25
**Size of keloids (cm** ^**2**^ **)**		
Mean	4.65	
Range	0.25–46.25	
**Activity administered (MBq)**		
Mean	256.7	
Range	35–663.50	
**Time of treatment (minutes)**		
Mean	350.89	
Range	85–1304	

### Follow-up

Patients were followed-up for a median period of 37 months (range: 11–59 months). There was a subjective response to therapy as documented on follow-up with patients reporting reduced pain, itching and reduced scar size. In all patients there was an initial phase (2 weeks post therapy) of erythema and ulceration followed by crusting and scab formation. There is a delayed phase of hypopigmentation in most patients. Complete response was documented in 43 (72%) of the lesions (Fig. 3), partial response in 6 (10%) lesions, progression in 4 lesions (7%) and stable disease in 6 lesions (10%) (Table 2).

**Table 2:** Lesion response per category

Response Category	Number of lesions	Initial average size	Average size reduction	Percentage total lesions
Complete response (> 70% reduction)	43	5.88cm^2^	4.71cm^2^	72.88%
Partial response (20–70% reduction)	6	9.27cm^2^	4.17cm^2^	10.17%
Progression (increase size and/ or height)	4	2.75cm^2^	0.41cm^2^	6.78%
No response / stable disease (< 20% reduction)	6	2.01cm^2^	0.20cm^2^	10.17%

## Discussion

In this work we report on our initial experience with ^188^Re using the Oncobeta system in patient with keloids. The use of radioactive material for superficial brachytherapy dates back to the 19th century when radium was used to treat cancers. Beta (β) emitters have been investigated as radionuclides in superficial brachytherapy. Vivante and colleagues explored the use of Phosphorus-32 (^32^P) embedded in a patch for the treatment of keloids and hypertrophic scars. They achieved responses ranging from partial remission with 50 − 90% reduction in size of the lesions to complete remission in other lesions [6]. Other authors have also reported on the use of radioactive patches for the treatment of superficial skin lesions [7–10]. Shukla et al. created customized ^188^Re patches which they applied on day one and three for 3 h each day. Most lesions required multiple sessions to achieve remission [11]. All lesions experienced symptomatic relief. Although patches can be effective, they have some drawbacks, including a complicated preparation process that exposes staff to radiation, and the therapy typically takes place over several days [11]. The applicator systems provides greater flexibility especially for lesions with complex geometries or larger surface areas. It also provides uniform distribution of the administered dose which results in personalized therapy [12–14]. Our study proves that superficial brachytherapy with ^188^Re is a great alternative for patients with keloids who may not be candidates for other therapies or in whom other therapies have proven unsuccessful.

We had complete response in 72% of our patients after a single session of therapy, which is similar to control rates of keloids achieved in various studies / therapies which range between 70 and 90% [15, 16]. Only 4/59 lesions demonstrated progression / recurrence. This is similar to the recurrence rates documented for radiation therapy with or without surgery or steroids which ranged from 4.7 to 29% [16–22]. Shukla and Mittal treated 15 keloid lesions with 188Re patches and noted a reduction in height and size in all lesions with complete flattening after the first therapy observed in small and / or newly formed keloid lesions [11]. No recurrences were noted during a 3- year follow-up period. Using ^188^Re embedded bandages, a group of authors reported on their findings of complete flattening / remission in one patient while there was an 80% reduction in the size of the lesions with marked flattening, in the other five patients [23]. These findings are encouraging and provide evidence that brachytherapy with ^188^Re is a viable option for the treatment of keloid patients with similar results to traditional therapies including radiation therapy. High-dose-rate (HDR) brachytherapy can cause damage to the DNA and inhibit angiogenesis in overactive keloids cells [24]. Radiation may be indicated for recurrent keloid lesions, unfavourable sites or in patients with high likelihood of recurrence [16]. Most radiation therapy procedures provide superior outcomes when the radiation is offered within the first 24–48 h post operatively. Although some of our patients had surgical histories, those procedures occurred long before their referral for ^188^Re treatment. Even in cases where debulking surgeries were needed, there was at least a month between the surgery and the ^188^Re therapy. This allows for flexibility in planning and scheduling of ^188^Re therapies.

Different radiation doses and schedules have been explored for keloid treatment, but there is no clear consensus on the best approach. Excision followed by 18 Gy of radiation therapy in three factions over 3 or 4 days showed recurrence rates of 0 − 19%, respectively [25, 26]. Other authors have investigated 10–15 Gy of radiation in varying fractions and frequencies at different time points with recurrence rates ranging between 3.1% and 24% [27–30]. The recommended total dose ranges from 12 to 20 Gy, however, new data suggests a dose greater than 30 Gy has better long term control [31]. Using ^188^Re patches, lower doses were used for the treatment of newly formed and small keloid lesions [32]. In our study we aimed to deliver 30 Gy to the deepest margin of the keloid lesion regardless of size and age, and we had a total recurrence rate of 7% while 10% of the lesions demonstrated stable disease. These results are in keeping with that of interstitial brachytherapy or external beam therapy. This dose seemed to provide a good compromise between side effects and therapeutic efficacy as evidenced by the non-inferior control rates. Direct comparisons of the administered dose, however, is challenging as there are major differences in study protocols including timing post-surgery, integration of other therapies such as intralesional steroid etc. and variable follow-up periods.

**Fig. 4:**
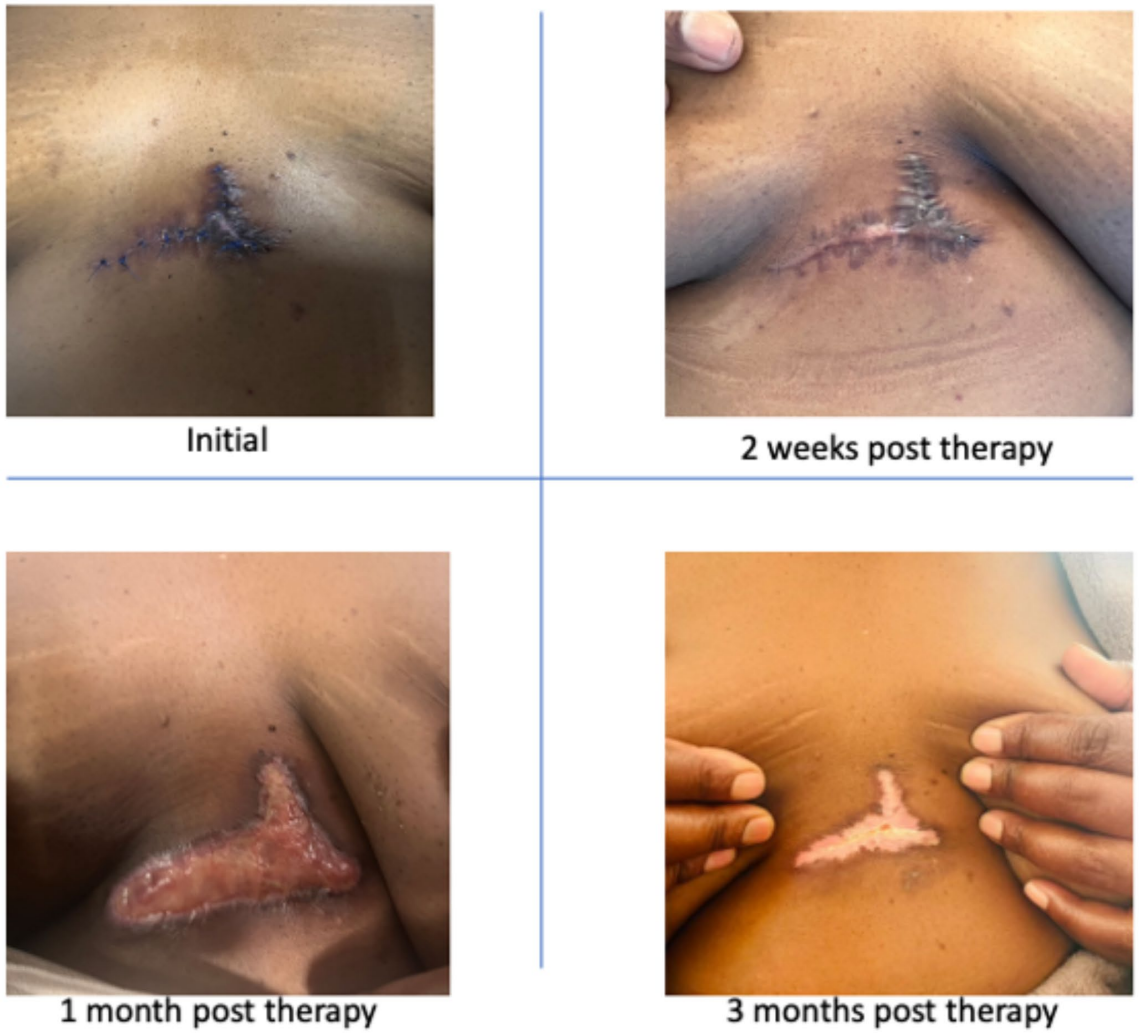
A 54 year old female with a chronic history of keloids. She complained of itching. She had received surgery 1 month prior to 188Re therapy. The administered activity was 250.5MBq with a total dose of 30 Gy. There was a complete response following 11 months of follow-up

The side effects that may be expected as a result of application of ^188Re^ include: skin redness, inflammation, bleeding, superficial vascular complications, local infections, skin necrosis, depigmentation and scar tissue. Most of the expected side effects are superficially on the skin, as there is no risk of incorporation of the ^188^Re into the body when applied according to the available methods or prescribed protocol. Most of the side effects related to the ^188^Re skin therapy are acute effects lasting only a few weeks while long term effect include hypopigmentation, alopaecia, hyperpigmentation or telangiectasia. While most of the published data on the use of ^188^Re as superficial brachytherapy has been on its use in skin cancers, the same principle applies for keloid therapy. Although, some authors reported no side effects [11, 12], Castelluci and colleagues reported on different grades of early skin toxicities which however resolved over 90 days with excellent cosmetic results [33]. Even with the use of patches, Shukla et al. also reported early changes including erythema, discharge of serum with scab formation and occasional bleeding. Late effects included skin discolouration, however, this was transient and subsided with time [11]. In our patients we also observed early skin toxicities such as erythema, oedema and ulceration which healed after ± 2 months. Most lesions resulted in hypopigmentation which is an expected side effect of the therapy (Fig. 4). Given the chronic and often recurrent nature of keloids, consistent and long-term follow-up protocols are essential for accurately assessing late toxicity following Rhenium-188 cream treatment. Keloids typically evolve over months to years, and delayed adverse effects may only become apparent after extended periods. Establishing standardized follow-up schedules will ensure comprehensive monitoring of any long-term skin reactions, fibrosis, or other potential toxicities associated with radiation therapy. This will be crucial in evaluating the safety profile of Rhenium-188 cream and ensuring its therapeutic benefit outweighs the risks in the long-term management of keloids.

Despite our modest study population, our findings on the use of Rhenium-188 cream in the treatment of keloids show promising results in improving clinical outcomes. However, to confirm and further validate these initial findings, large multicentre studies are essential. These studies will provide a broader patient population, enhancing the generalizability of the results, and allow for a more robust analysis of the dose-response relationship. By incorporating varied treatment protocols, patient demographics, and lesion characteristics, future trials will refine optimal dosing strategies, enabling us to determine the most effective and safe therapeutic regimens for keloid management using Rhenium-188.

We also had irregular follow-up schedules with telephonic follow-up of some patients. Our follow-up could be improved by the addition of more subjective measures of therapy response such as the use of quality of life questionnaires. This is an important part of the overall management as keloids have a huge impact on the psychosocial aspect of patients. This was incorporated late in our protocol, therefore not all patients completed the questionnaires and there was insufficient data to make analyses.

## Conclusion

Keloids often have an intractable course with recurrences regardless of the available treatment options. Early work on the use of radionuclides for the treatment of keloids has shown encouraging results. In this work we have demonstrated the feasibility of using ^188^Re for the treatment of keloids in select cases. The use of the specialized applicator system offers ease of use and great flexibility in delivering the desired radiation dose and great clinical and cosmetic results. In cases with bulky lesions, we recommend a combined approach of initial surgery to reduce the mass followed by application of ^188^Re ± 1 month post-surgery (when the wound has healed / stitches removed). While the initial results are promising, it is important to recognize that these findings are based on a small cohort and require further validation. Thus, clinical trials are warranted to assess the efficacy of ^188^Re as monotherapy or in combination with surgery to guide the clinical utility of this therapy in keloids.

## Data Availability

The datasets generated during and/or analysed during the current study are available from the corresponding author on reasonable request.
